# Feasibility and Safety of Post-Transcatheter Aortic Valve Replacement Coronary Revascularization Guided by Stress Cardiac Imaging

**DOI:** 10.3390/jcm13195932

**Published:** 2024-10-05

**Authors:** Florence Leclercq, Mariama Akodad, Elvira Prunet, Fabien Huet, Pierre-Alain Meunier, François Manna, Jean-Christophe Macia, Pierre Robert, Matthieu Steinecker, Jean-Michel Berdeu, Laurent Schmutz, Thomas Gandet, François Roubille, Guillaume Cayla, Denis Mariano-Goulart, Benoît Lattuca

**Affiliations:** 1 Cardiology Department, Arnaud de Villeneuve University Hospital, University of Montpellier, 34293 Montpellier, France; akodadmyriam@gmail.com (M.A.); huetfabienmontp@gmail.com (F.H.); pierrealain.meunier@gmail.com (P.-A.M.); jc-macia@chu-montpellier.fr (J.-C.M.); matthieu.steinecker@chu-montpellier.fr (M.S.); jean-michel.berdeu@chu-montpellier.fr (J.-M.B.); francois.roubille@gmail.com (F.R.); 2 Cardiology Department, Caremeau University Hospital, Montpellier University, 30900 Nîmes, France; elviraprunet@gmail.com (E.P.); pierrecardio@gmail.com (P.R.); laurent.schmutz@chu-nimes.fr (L.S.); cayla.guillaume@gmail.com (G.C.); benoit.lattuca@gmail.com (B.L.); 3 Department of Epidemiology, Medical Statistics and Public Health, Arnaud de Villeneuve University Hospital, 34090 Montpellier, France; f-manna@chu-montpellier.fr; 4 Department of Cardiac and Thoracic Surgery, Arnaud de Villeneuve Hospital, 34090 Montpellier, France; t-gandet@chu-montpellier.fr; 5 Department of Nuclear Medicine, University Hospital of Montpellier, 34295 Montpellier, France; d-mariano_goulard@chu-montpellier.fr

**Keywords:** aortic stenosis, TAVR, coronary artery disease, percutaneous coronary intervention, myocardial ischemia

## Abstract

**Background:** Systematic revascularization of asymptomatic coronary artery stenosis before transcatheter aortic valve replacement (TAVR) is controversial. **Purpose:** The purpose of this study was to evaluate the feasibility and safety of functional evaluation of coronary artery disease (CAD) followed by selective ischemia-guided percutaneous coronary revascularization following TAVR. **Methods:** This prospective, bi-centric, single-arm, open-label trial included all patients with severe aortic stenosis (AS) eligible for TAVR and with significant CAD defined as ≥1 coronary stenosis ≥ 70%. Patients with left main stenosis ≥ 50%, proximal left anterior descending artery (LAD) stenosis ≥ 90% or > class 2 Canadian Classification Society (CCS) angina were excluded. Myocardial ischemia was evaluated by stress cardiac imaging one month after TAVR. The primary endpoint was a composite of all-cause death, stroke, major bleeding (Bleeding Academic Research Consotium ≥ 3), major vascular complication (Valve Academic Research Consortium 3 criteria), acute coronary syndrome (ACS) and hospitalization for cardiac causes within 6 months of receiving TAVR. **Results:** Between June 2020 and June 2022, 64 patients were included in this study. The mean age was 84 ± 5.2 years. CAD mostly involved LAD (n = 27, 42%) with frequent multivessel disease (n = 30, 47%) and calcified lesions (n = 39, 61%). Stress cardiac imaging could be achieved in 70% (n = 46) of the patients, while 30% (n = 18) did not attend the stress test. Significant myocardial ischemia was observed in only three patients (4.5%). At 6-month follow-up, fifteen patients (23%) reached the primary endpoint, including death in six patients (9%), stroke in three patients (5%) and major bleeding in three patients (5%). ACS was observed in only two patients (3%) but both had severe coronary stenosis (≥90%) and did not refer for stress imaging for personal reasons. Hospital readmission (n = 27, 41%) was mostly related to non-cardiac causes (n = 17, 27%). **Conclusions:** In patients with asymptomatic CAD scheduled to undergo TAVR, a selective ischemia-guided coronary revascularization after TAVR seems to be safe, with a very low rate of ACS and few cases of myocardial ischemia requiring revascularization, despite low adherence to medical follow-up in this elderly population. This strategy could be evaluated in a randomized study.

## 1. Introduction

Observational studies revealed a prevalence of coronary artery disease (CAD) associated with severe aortic stenosis (AS) in approximately 40 to 60% of patients [1,2,3]. CAD has been associated with impaired long-term prognosis, and current guidelines state that myocardial revascularization at the time of surgical aortic valve replacement is a class I recommendation in the presence of angiographically significant coronary artery stenosis [4,5]. Conversely, the management of concomitant CAD in patients with severe AS undergoing transcatheter aortic valve replacement (TAVR) is still a matter of debate [6,7]. While reduced left ventricular ejection function (LVEF) and comorbidities are associated with adverse events after TAVR, CAD was not systematically associated with impaired outcomes [8,9,10]. By extrapolation with surgery, the strategy of systematic coronary angiography and percutaneous coronary intervention (PCI) according to angiographic evaluation only, has been proposed to patients undergoing TAVR, without a demonstrated benefit and with evidence only based on observational or retrospective studies [9,10,11]. While systematic PCI is usually proposed before TAVR in proximal and tight coronary lesions, the randomized ACTIVATION study showed no benefit to systematic revascularization of coronary artery stenosis by PCI before TAVR [12]. Conversely, in the recent NOTION- 3 study, PCI was associated with a lower risk of a composite of death from any cause, myocardial infarction or urgent revascularization at a median follow-up of 2 years than conservative treatment [13].

While coronary revascularization in patients with asymptomatic CAD is controversial in the absence of significant ischemia [14], the functional evaluation of induced myocardial ischemia is challenging in severe AS [15,16]. Although systematic PCI in case of AS has not been associated with higher mortality rates [10,11,16], it may result in procedural complications such as acute kidney failure, stent thrombosis, coronary dissection, vascular access complications or major bleeding, particularly among the old and frail population of TAVR patients.

Thus, we aimed to evaluate the feasibility and safety of a non-systematic but selective ischemia-guided coronary revascularization among patients with asymptomatic CAD referred for TAVR. A delayed evaluation of myocardial ischemia using cardiac stress imaging after removing the aortic hemodynamic obstacle would allow for the identification of significant coronary artery stenosis and the performance of selective PCI. 

## 2. Methods

### 2.1. Study Oversight 

The REVASC-TAVR trial is a bi-centric, phase 2, prospective, single-arm, open-label, investigator-initiated trial conducted at the cardiology department of Montpellier and Nimes University Hospitals (France). 

The study protocol was approved by an independent ethics committee before study initiation (Comité de Protection des Personnes Sud Méditerranée, Montpellier, France, ID RCB: 2015-A01823-46) and all patients provided oral and written informed consent providing information about the strategy. This study is registered in the Institutional Review Board (IRB number: 9674). An independent and out-of-region located safety monitoring committee oversaw this study. The trial was conducted according to the World Medical Association Declaration of Helsinki and was registered with ClinicalTrials.gov (NCT02797158). 

### 2.2. Patient Population

All patients included were older than 18 years and were admitted for pre-operative evaluation before TAVR for the treatment of severe and symptomatic AS diagnosed by transthoracic echography (TTE). All patients had significant CAD defined as follows: ≥1 stenosis of ≥70% in a major epicardial coronary artery with a diameter > 2.5 mm, excluding left main stenosis > 50% or proximal left anterior descending artery (LAD) stenosis > 90%. Only patients without severe angina symptoms were included (≤ class 2 Canadian Cardiovascular Society (CCS) angina) [17]. The patients were not eligible for surgical aortic valve replacement after evaluation by the local heart team, including at least an interventional cardiologist, a cardiothoracic surgeon and a gerontologist. All the eligibility criteria are summarized in Table 1. 

### 2.3. Study Design

**Figure 1:**
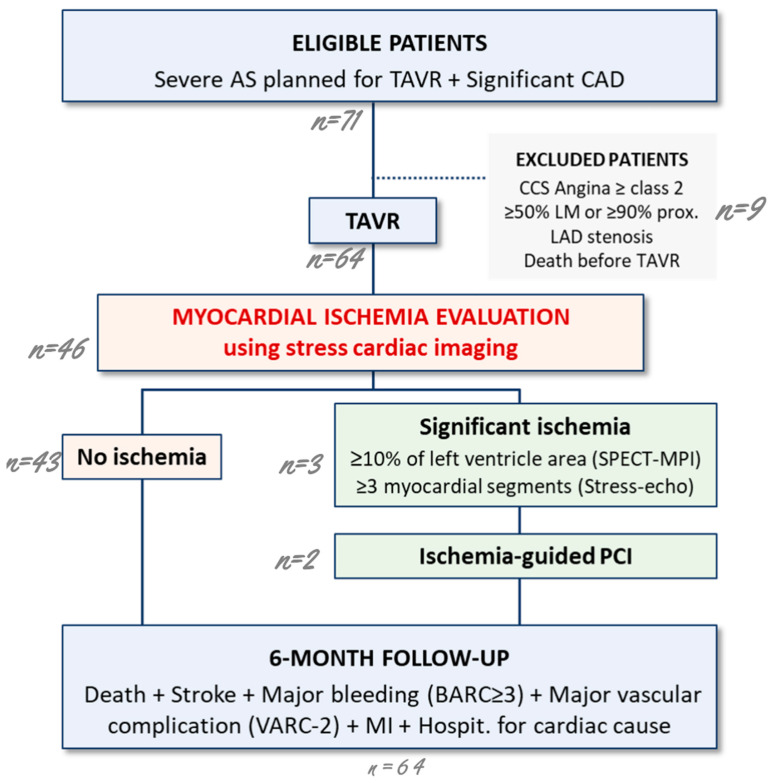
Design of the trial. AS means aortic stenosis; BARC, Bleeding Academic Research Consortium; CAD, coronary artery disease; LAD, left anterior descending artery; LM, left main; MI, myocardial infarction; SPECT-MPI, Single-photon emission computed tomography–myocardial perfusion imaging; TAVR, transaortic valve replacement; VARC, Valve Academic Research Consortium.

The inclusion was performed after initial coronary angiography. A functional evaluation of myocardial ischemia using single-photon emission computed tomography–myocardial perfusion imaging (SPECT MPI) or stress echocardiography was scheduled one month after TAVR for all patients. A PCI was only performed in the presence of significant myocardial ischemia as recommended by current guidelines in stable CAD [7,14]. Follow-up visits were scheduled with a cardiologist at 1 and 6 months following TAVR and included clinical examination, ECG, echocardiography and reporting of all the events as described in the study endpoints. The follow-up was completed by phone contact (patient or referent doctor) if necessary. The study design is presented in Figure 1. 

### 2.4. Transcatheter Aortic Valve Replacement (TAVR)

The procedure was performed using balloon-expandable Edwards SAPIEN 3 prosthesis (Edwards Lifesciences, Irvine, CA, USA) or self-expandable CoreValve Evolut R or Evolut Pro prosthesis (Medtronic, Inc., Minneapolis, MN, USA). All procedures were performed under local anesthesia with sedation in a hybrid operating room with the presence of at least an interventional cardiologist, a cardiothoracic surgeon and an anesthesiologist. Vascular access was mainly percutaneous.

### 2.5. Stress Cardiac Imaging

Functional evaluation was performed one month following TAVR and was based on single-photon emission computed tomography–myocardial perfusion imaging (SPECT-MPI) or stress echocardiography using dobutamine perfusion depending on patient characteristics and at the discretion of the physicians. 

Concerning SPECT-MPI, a one-day protocol was performed including a rest SPECT MPI acquisition followed by stress SPECT MPI acquisition. After a resting time, patients received 2.7 MBq/kg of 99 mTc (tetrofosmin) intravenously before being installed under a cardiac-dedicated multi-pinhole CZT camera. Patients received an intravenous injection during 4 min of 0.56 mg/kg of dipyramidole and, if they were able to make effort, a combined maximal exercise stress test on a treadmill was performed [18]. Ischemia was defined as a reversible perfusion defect and necrosis as a non-reversible defect. Ischemia was considered significant when more than 10% of the left ventricle was involved.

Stress echocardiography was based on intravenous infusion of dobutamine in 3 min increments, starting with 5 µg/kg/min and increasing to 10, 20, 30 and 40 µg/kg/min. If necessary to reach at least 85% of the maximal heart rate, atropine (in a dose of 0.25 mg up to a maximum of 1 mg) was added to the dobutamine infusion. An ischemic response was defined as a worsening of a regional wall function at stress and a necrotic response as a fixed resting dysfunction during stress. A myocardial ischemia was considered significant if ≥3 myocardial segments were involved [19].

### 2.6. Percutaneous Coronary Intervention

When significant ischemia was detected, coronary angiography control and ischemia-guided PCI were performed. All patients received an oral loading dose of 600 mg of clopidogrel in association with 250 mg of intravenous aspirin and an appropriate intravenous anticoagulant therapy: 70–100 U/kg of unfractionated heparin (UFH) or 0.5 mg/kg of intravenous enoxaparin. Radial access was the preferred approach, and the PCI technique was at the discretion of the interventional cardiologist according to international guidelines. Only drug-eluting stents were used, and a dual antiplatelet therapy including 75 mg of aspirin and 75 mg of clopidogrel was prescribed at discharge and recommended during 6 months, except in case of high bleeding risk according to guidelines [14]. 

### 2.7. Study Endpoints

The primary endpoint was the composite of all causes of death, stroke, major bleeding (Bleeding Academic Research Consortium (BARC) ≥ 3) [20], major vascular complication (Valve Academic Research Consortium 3 criteria) [21], myocardial infarction (MI) and hospitalization for cardiac causes at 6 months following TAVR. Hospitalization for cardiac causes was described as related to heart failure, acute coronary syndrome or rhythmic/conductive disorders. The secondary endpoints included evaluation of the individual components of the primary endpoint at 30 days and 6 months after TAVR. The occurrence of cardiovascular death, any coronary revascularization, all causes of hospitalization and cardiac conductive disorders requiring permanent pacemaker implantation were also evaluated at the same time points. Periprocedural complications (ventricular fibrillation (VF), ventricular tachycardia (VT) requiring cardioversion, cardiorespiratory arrest requiring cardiopulmonary resuscitation (CPR) or assisted mechanical ventilatory support) were also assessed.

### 2.8. Statistical Analysis and Sample Size Determination

Our hypothesis was that delayed ischemia-guided coronary revascularization would be associated with a similar rate of clinical outcomes as the conventional strategy of systematic coronary revascularization before or during TAVR. Based on previous studies, a rate of 40% of clinical events at 6-month follow-up was considered in a population of patients admitted for TAVR with concomitant CAD [8,11,22,23],. Thus, considering a maximal rate of 40% clinical outcomes, the inclusion of 62 patients, of whom 19 patients would meet the primary endpoint, allowed for a statistical power of 80%. Considering a maximal discontinuation of 15% of the patients, the inclusion of 71 patients was required. Descriptive analysis and quantitative continuous variables were presented using mean values, quartiles and standard deviations. Qualitative categorical variables were described using frequencies and proportions. A *p*-value of 0.05 was used for statistical significance. The data were analyzed by the statistical department of Montpellier University Hospital.

## 3. Results

### 3.1. Study Population

Of 71 eligible patients, 64 patients were included in the analysis and completed the follow-up. Five patients were excluded because they did not comply with the study protocol and two patients died before TAVR, one from a non-cardiovascular cause and one from heart failure related to severe left ventricular dysfunction. The population included a majority of men, with a mean age of 84 ± 5.2 years, and 31% (n = 20) of the patients had previous ischemic heart disease. Baseline characteristics and echocardiographic parameters are fully presented in Table 2. Coronary artery disease mostly involved LAD (n = 27, 42%) and was severe, with frequent multivessel disease (n = 30, 47%) and more than half of the patients with calcified lesions (n = 39, 61%). (Table 3). The mean delay between initial coronary angiography and TAVR was 15 ± 8 days. TAVR was mainly performed by the percutaneous approach (n = 39, 61%) and femoral access (n = 47, 73%). The Edwards SAPIEN 3 valve was mainly implanted (n = 42, 66%).

### 3.2. Functional CAD Evaluation

Stress cardiac imaging was performed in 46 patients (72%) with a mean delay of 38 ± 12 days after the TAVR procedure. A significant number of patients (n = 18, 28%) did not refer to the stress cardiac imaging appointment, mainly due to a secondary impaired medical condition (n = 7, 11%) or patient refusal (n = 7, 11%). Three patients had significant myocardial ischemia (between 10 and 15% of the inferior and lateral or the inferior territory) and in all of them, ischemia was related to coronary artery stenosis (right coronary artery). Finally, two of them had successful PCI. The third patient had a chronic total occlusion managed medically.

### 3.3. Clinical Outcomes

At 6 months, 15 patients (23%) met the primary endpoint, which was mainly related to hospitalization for cardiac causes (n = 9, 14%), death (n = 6, 9%) or major bleeding (BARC ≥ 3) (n = 3, 5%). Death was mainly related to non-cardiovascular causes (n = 3, 5%) and only one patient presented with fatal cardiogenic shock following TAVR. Five out of six deaths occurred among patients who did not perform the stress cardiac imaging. The three major bleeding complications were related to one local thoracic hematoma following pacemaker implantation, one pericardial tamponade secondary to a temporary pacemaker used during TAVR and one femoral hematoma.

Hospitalizations for cardiac causes during follow-up occurred in nine patients (14%) and were mainly related to syncope or conductive disorders and heart failure (Table 4). Only two patients had acute coronary syndrome related to a culprit lesion associated with >90% stenosis in a proximal coronary artery (respectively, right coronary artery and left circumflex artery) identified during the coronary angiography before TAVR. Stress cardiac imaging had not been performed in these patients, in the former because ACS occurred before the scheduled stress test and the latter due to a worsening of their general condition resulting in patient refusing to perform the test.

Hospitalization for non-cardiovascular causes occurred in 21 patients (33%) and was mainly related to fractures and orthopedic injuries (n = 4, 6%), cancer (n = 4, 6%), sepsis (n = 4, 6%) and anemia or digestive bleeding (n = 3, 5%).

Eleven patients presented with a cardiovascular event between the initial coronary angiography and TAVR: two patients died and were excluded from the follow-up and nine patients presented with acute heart failure leading to hospitalization, but none of them had an acute coronary syndrome.

## 4. Discussion

The therapeutic management of patients with significant CAD scheduled for TAVR is challenging and the meaningful issue of the indication and optimal timing for coronary revascularization remains controversial. This study aimed to evaluate a strategy of delayed coronary revascularization guided by a functional evaluation after TAVR and reports three main findings. First, a strategy of non-systematic but selective coronary revascularization with PCI following TAVR appears safe, as major cardiovascular events at follow-up were frequent in this high-risk population but were mainly related to non-cardiovascular- and non-coronary-related events. Second, the incidence of myocardial ischemia was low in this population with asymptomatic and stable CAD. Third, the evaluation of CAD by a functional test in elderly and often frail patients appears difficult in everyday clinical practice, considering one-third of patients did not perform the test in our study for medical reasons or due to patient decision.

The definition of significant CAD among patients undergoing TAVR is not standardized, resulting in a prevalence of CAD between 40 and 75% reported in studies and in a controversial impact of CAD on patient prognosis [7,8,9,10,11]. Previous PCI or CABG was associated with increased mortality but was often associated with poor prognostic factors such as a high-risk SYNTAX score, high Euroscore or reduced LVEF [10,21,24,25]. The timing of performing PCI in case of significant CAD has also been debated [6,7,11]. The safety of PCI in case of AS was demonstrated in several studies, without an increase in mortality rate after revascularization [9,10,11,23]. However, performing TAVR early after PCI may increase the risk of postprocedural bleeding related to complications of vascular access or acute kidney injury associated with contrast delivery. Conversely, performing PCI after TAVR with self-expandable bioprothesis, may be challenging due to the stented frame placed over the coronary ostia making selective coronary catheterization difficult [7,11,16]. Whatever the chosen strategy, CAD is often diffuse and severe in the elderly, as demonstrated in our study, with frequent multivessel disease and with more than half of patients with calcified lesions. Such coronary status could increase the risk of PCI complications, particularly in elderly patients. Moreover, the benefit of systematic coronary revascularization on major adverse cardiac events in stable patients is not proved [7,8,9,10,11,23,26], particularly in calcified lesions, which are usually very stable and frequently associated with slow evolution.

The majority of events in the elderly and frail TAVR patients in our study were mainly related to non-cardiovascular and non-coronary events, as previously described [8,27,28]. A strategy of selective and ischemia-guided PCI appears safe, with a very low incidence of acute coronary syndrome at 6 months (3%), which is consistent with previous studies evaluating systematic revascularization before TAVR [9,16]. These results highlight that myocardial ischemia associated with CAD remains rare in asymptomatic or stable CAD patients referred for TAVR. Our study also suggests that a delayed PCI does not increase the risk of acute coronary events after TAVR. Interestingly, while patients with severe LAD stenosis were excluded from our study, the only two acute coronary syndromes reported were related to severe and proximal circumflex and tight right coronary artery stenosis. Moreover, these two patients did not undergo a functional test, which could have detected myocardial ischemia and possibly prevented the acute coronary event. Consistent results were recently published and support a reasonably incomplete revascularization according to heart team decision, suggesting that only proximal and very tight coronary stenosis (≥90%) may be associated with an increasing risk of coronary events and should be systematically treated [29].

Current guidelines recommend performing PCI only after the evaluation of myocardial ischemia assessed by a functional test in asymptomatic patients presenting with significant CAD [14]. In the case of AS, an appropriate evaluation of myocardial ischemia related to coronary artery stenosis may be difficult [15]. Myocardial hypertrophy is associated with a reduction in the vasodilatory reserve of the coronary circulation, leading to a challenging evaluation of Fractional Flow Reserve (FFR) [15,30]. Functional testing by non-invasive stress cardiac imaging techniques are also not recommended in case of severe AS due to the significant hemodynamic obstacle and the risk of significant decrease in the cardiac output leading to syncope [15]. Recent studies suggested, however, that functional invasive and non-invasive evaluation of CAD could be possible before TAVR. However, despite the increased interest in understanding coronary physiological changes caused by aortic stenosis, data on the evaluation of coronary hemodynamics in that setting are still scarce [31,32]. Our study is the first to evaluate myocardial ischemia after TAVR using a stress cardiac imaging modality and aiming to consider coronary patients after TAVR to be usual patients with CAD. However, the assessment of myocardial ischemia was not optimal, and functional evaluation was performed in only 70% of our population, mainly due to the worsening of patients’ clinical condition and intercurrent non-cardiovascular events in our elderly population. This finding emphasizes the difficulty of performing a systematic functional evaluation in elderly patients with several comorbidities.

The randomized controlled ACTIVATION trial showed that PCI prior to TAVR in patients with significant CAD produced noninferior clinical results (death and rehospitalization at 1-year follow-up) when compared with medical treatment, with more bleeding events in the PCI arm [12]. In the NOTION-3 study, PCI was associated with a lower risk of a composite of death from any cause, myocardial infarction or urgent revascularization and the results suggested that the benefit of PCI appeared to be greatest in lesions with a diameter stenosis of at least 90%, suggesting the impact of myocardial ischemia with the risk of adverse events [13]. Interestingly, in these two studies, the control arm is a medical treatment strategy only without evaluation of myocardial ischemia as proposed in our study. Considering that only patients with very tight (≥90%) and proximal stenosis had acute coronary events in our study, systematic PCI may be considered in these patients, while all other coronary lesions (non-proximal stenosis, significant but not severe coronary stenosis, coronary vessels diameter < 2.5mm) could be medically treated and secondarily evaluated by stress test after TAVR, as in usual patients with CAD.

With the extension of TAVR indication in lower-risk patients, the management of CAD in TAVR patients requires long-term follow-up. While awaiting further data to provide strong recommendations, the decision to treat significant coronary lesions in TAVR patients should be individually managed based on clinical and anatomical factors according to the local heart team’s expertise [6,7,26].

The present work has to be considered in the light of the following limitations. First, our study was a bi-centric, non-randomized study with relatively small sample size. Second, a cardiac stress test was not performed in one-third of our study population, which does not allow us to draw conclusions for these patients.

## 5. Conclusions

In elderly patients with significant coronary disease scheduled for TAVR, prognostic and adverse outcomes are mainly related to non-cardiovascular events. A strategy of non-systematic but selective ischemia-guided coronary revascularization appears safe, with a very low risk of acute coronary events and myocardial ischemia requiring revascularization, only related to proximal and very tight coronary stenosis. Systematic PCI of these specific lesions may be considered while all other coronary lesions could be medically treated and secondarily evaluated by stress test after TAVR according to individual global clinical condition of each patient. In that setting, non-invasive functional evaluation of CAD before TAVR is probably promising. Large-scale and randomized trials are, however, warranted to validate future strategies of ischemia evaluation of CAD in aortic stenosis.

## Figures and Tables

**Table 1:** Study eligibility criteria.

Inclusion Criteria	Definitions
Patients > 18 years	
Severe aortic stenosis	- Mean transaortic gradient ≥ 40 mmHg - Aortic valve area ≤ 1.0 cm^2^
Symptoms suggestive of severe aortic stenosis	- Dyspnea - Syncope - Angina but with CCS ≤ 2
Patient eligible for TAVR after heart team evaluation	
Significant coronary artery disease	≥1 stenosis of ≥70% in a major epicardial coronary artery with a diameter > 2.5 mm (except for left main or proximal left anterior descending artery)
Written informed consent	
Insured under the French security health system	
Exclusion criteria	Definitions
Recent acute coronary syndrome	Within 30 days before inclusion
Stenosis > 50% of the left main or >90% of the proximal LAD	
Significant angina	CCS class > 2
Aortic regurgitation III or IV	
Active bleeding	
Contra indication for tomographic technetium-99 assessment or dipyridamole injection	- Acute coronary syndrome within 5 days - Unstable angina - Refractory heart failure - Refractory severe arrhythmia - Arterial hypotension (systolic blood pressure < 90 mmHg) - Asthma - Severe pulmonary arterial hypertension - Bradycardia or severe cardiac conduction disorders - Dipyramidole allergy - Chronic obstructive pulmonary disease - Stroke within 30 days
Contraindication to local anesthesia	
Previous enrolment in another study	
Impossibility to obtain written consent	

CCS means Canadian Cardiovascular Society classification; LAD: Left Anterior Descending.

**Table 2:** Baseline patient characteristics.

Variables	n = 64
**General parameters**	
Male gender	38 (59)
Age (years)	84 ± 5.2
Body mass index (kg/m^2^)	26 ± 4.6
**Cardiovascular risks factors**	
Hypertension	48 (75)
Diabetes	17 (27)
Dyslipidemia	23 (36)
Active smoking	4 (6)
**Comorbidities and medical past history**	
Ischemic heart disease	20 (31)
Stroke	5 (8)
Peripheral arterial disease	9 (14)
Chronic respiratory failure	8 (13)
Atrial fibrillation	23 (36)
Pacemaker	6 (9)
Chronic kidney disease	23 (36)
**Logistic Euroscore**	13 ± 8.6
**Euroscore 2**	3.9 ± 5.3
**Symptoms at inclusion**	
NYHA I or II	25 (39)
NYHA III or IV	39 (61)
Angina (CCS < 2)	5 (8)
Syncope	3 (5)
**Chronic treatment**	
Statins	36 (56)
Betablockers	32 (50)
ACEI	21 (33)
Aspirin	38 (59)
Clopidogrel	8 (13)
Anticoagulation therapy (VKA or DOA)	16 (25)
**Echocardiographic parameters**	
Left ventricular ejection fraction (%)	52 ± 12
Max transaortic gradient (mmHg)	74 ± 20
Mean trans aortic gradient (mmHg)	49 ± 12
Max trans aortic velocity (m/s)	4.3 ± 0.7
Aortic area (cm^2^)	0.7 ± 0.2
Aortic regurgitation III or IV	0 (0)
Mitral regurgitation stage III or IV	4 (6)

Values are numbers and percentages or numbers and standard deviations. ACEI means angiotensin-converting enzyme inhibitor; CCS, Canadian Cardiovascular Society classification; DOA, direct anticoagulant therapy, NYHA, New York Heart Association classification; VKA, vitamin K antagonist.

**Table 3:** Coronary angiography characteristics.

Variables, n (%)	n = 64
**Localization of the coronary artery stenosis**	
LAD, n (%)	27 (42)
Diagonal branch, n (%)	18 (28)
Left circumflex artery, n (%)	17 (27)
Left marginal artery, n (%)	15 (23)
Right coronary artery, n (%)	26 (41)
Posterior interventricular or retroventricular artery, n (%)	4 (6)
**Multivessel coronary disease, n (%)**	30 (47)
**Type of artery lesion**	
Chronic total occlusion, n (%)	8 (13)
Calcified lesion, n (%)	39 (61)
Tortuous lesion, n (%)	3 (5)
Bifurcation, n (%)	17 (27)
B2/C * lesion, n (%)	16 (25)

LAD means left anterior descending artery. * Complex lesion according to the ACC/AHA coronary lesion classification.

**Table 4:** Clinical outcomes at 6 months.

Variables, n (%)	n = 64
Combined endpoint	15 (23)
Death From any cause, n (%) From cardiovascular cause	6 (9)
Stroke, n (%)	1 (2)
Major bleeding, n (%)	3 (5)
Major vascular complication, n (%)	1 (2)
Acute coronary syndrome, n (%)	2 (3)
Hospitalization for cardiac causes, n (%)	9 (14)
Hospitalization for non-cardiovascular causes, n (%)	21 (3)

## Data Availability

Data are contained within article.
